# Functional Classification Using Phylogenomic Inference

**DOI:** 10.1371/journal.pcbi.0020077

**Published:** 2006-06-30

**Authors:** Duncan Brown, Kimmen Sjölander

**Affiliations:** Whitehead Institute, United States of America

Phylogenomic inference of protein (or gene) function attempts to address the question, *“What function does this protein perform?”* in an evolutionary context. As originally outlined by Jonathan Eisen [1–3], phylogenomic inference of protein function is a multistep process involving selection of homologs, multiple sequence alignment (MSA), and phylogenetic tree construction; overlaying annotations on the tree topology; discriminating between orthologs and paralogs; and—finally—inferring the function of a protein based on the orthologs identified by this process and the annotations retrieved. Figure 1 shows an example of using annotated subfamily groupings to infer function, in a manner similar to [1]. One of us, while at Celera Genomics, separately came up with a similar approach for the functional classification of the human genome [4], based on the automated identification of functional subfamilies using the SCI-PHY algorithm and the use of subfamily hidden Markov models (HMMs) to classify novel sequences [5,6]. Our experiences over the past several years in developing computational pipelines for automating phylogenomic inference at the genome scale [7]—and the challenges we have faced in this effort—motivate this paper.

**Figure 1:**
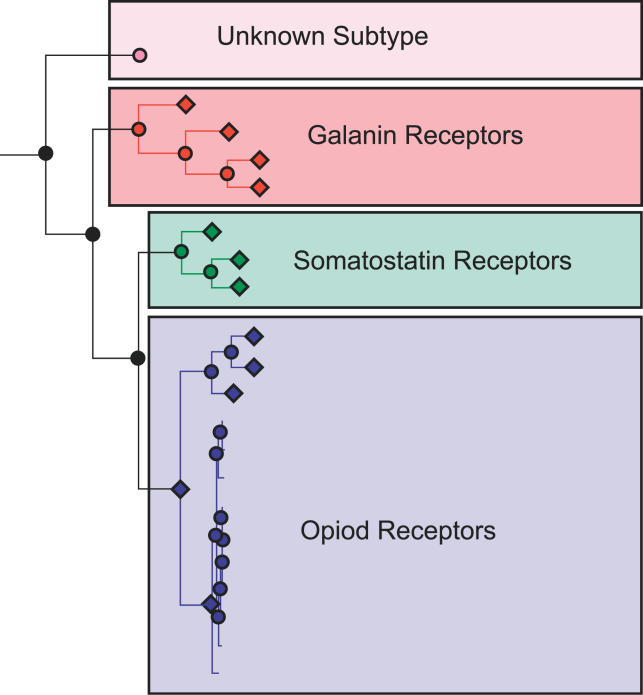
Phylogenomic Analysis of Protein Function Using Subfamily Annotation In the example shown above, a phylogenetic tree has been constructed for a set of G protein–coupled receptors. The molecular function of some of the members of the family has been determined experimentally and is used to annotate individual subfamilies, similar to [1]. Sequences without known function can be assigned a predicted molecular function using the tree topology to identify orthologs. When no experimental evidence is available for a subtree's molecular function (e.g., the *Unknown Subtype* subtree at top), the annotation would be left at a general level (e.g., “GPCR of unknown specificity, related to opioid, galanin, and somatostatin receptors”). By contrast, if the *Unknown Subtype* subtree were nested within a subtree whose members were consistently characterized, such as opioid receptors, a “subtree neighbors” approach could be used to assign the annotation “Putative opioid receptor” to that group [14]. The use of subfamilies as the basis of phylogenomic inference is only one approach; as noted in the text, the general methodology does not rely on subfamily groupings and would ideally use the entire tree topology.

In practice, phylogenomic inference of gene function is not often used. Far from it. The majority of novel sequences are assigned a putative function through the use of annotation transfer from the top hits in a database search. In our analysis of over 300,000 proteins in the UniProt database, only 3% of proteins with informative annotations (i.e., those not labelled as “hypothetical” or “unknown”) had experimental support for their annotations; 97% were annotated using electronic evidence alone. These annotations are uploaded to GenBank, where they persist even if they are eventually determined to be in error.

The systematic errors associated with this annotation protocol have been pointed out by numerous investigators over the years [8–10]. The root causes of these errors are these:

Gene duplication. This enables protein superfamilies to innovate novel functions on the same structural template, so that the top database hit may have a function distinct from the query.

Domain shuffling. Domain fusion and fission events add an additional layer of complexity, as a query and database hit may share only a local region of homology and thus have entirely different molecular functions and structures.

*Propagation of existing errors in database annotations*. This is particularly pernicious, as existing annotation errors are seldom detected and, even if detected, are not necessarily corrected.

Evolutionary distance. Two proteins can share a common ancestor and domain structure, yet have very different functions simply due to their presence in very divergently related species.

Phylogenomic analysis, properly applied, avoids these errors and provides a mechanism for detecting existing database annotation errors [3,7]. Why then is phylogenomic inference not used more widely? We believe this is due to four reasons. First, the actual frequency of annotation error is not known, so the gravity of the situation is not recognized. Second, phylogenomic inference is a much more complicated endeavor than a simple database search and requires significantly more expertise and computing resources. It is therefore not easily applied at the genome scale. Third, millions of dollars and years of effort have been poured into developing computational annotation systems that depend on annotation transfer from top database hits, perhaps overlaid with domain prediction methods such as PFAM or the NCBI CDD [11,12]. Fourth, phylogenomic approaches to protein function prediction have arisen only in the last few years, while database search methods have been available for much longer. Revolutions do not normally take place overnight. These four reasons result in phylogenomic inference being applied on a one-off basis, for a few protein superfamilies here and there.

This may be about to change. A variety of software tools and algorithms enabling phylogenomic inference have been developed in recent years (see Table 1). Some of these methods have based annotation transfer on the identification of orthologs [13–15] or of functional subfamilies [6,16–21]. Other groups have used whole-tree analyses [22–24]. Still other groups employ expert knowledge to define functional subtypes and then develop statistical models to allow users to classify novel sequences [25,26]; these expert system-based approaches are unfortunately limited by the scarcity of experimental data for most protein families.

**Table 1:**
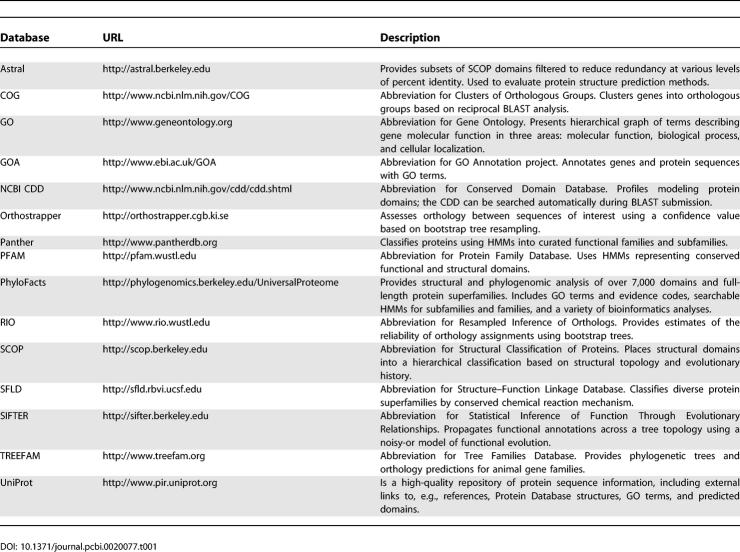
Resources for Phylogenomic Analysis

It is worth examining the assumptions underlying these phylogenomic resources, and phylogenomic inference as a whole.

## Tree Topology Accuracy

Phylogenomic inference is based on a fundamental assumption: the phylogenetic tree topology used as the basis of functional inference is correct. This assumption must be questioned, particularly when highly divergent sequences (e.g., with pairwise identities less than 25%) are included in a tree.

**Figure 2:**
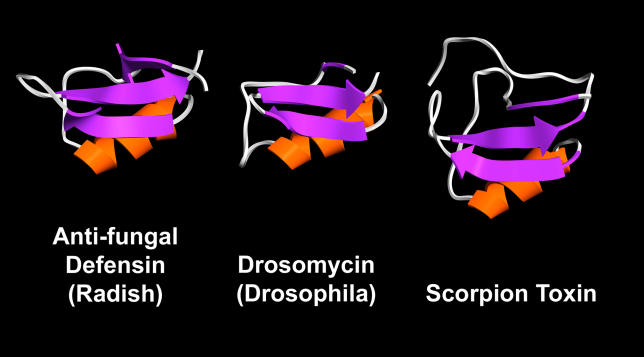
Structural and Functional Differences in Distantly Related Protein Superfamilies The three proteins shown above are all members of the Structural Classification of Proteins (SCOP) scorpion toxin–related superfamily. All retain the same basic fold, but have significantly divergent functions. They function as part of the innate immune arsenal in plants and insects, but form part of the offense in scorpions. Evolution has conserved the basic structure, but many residues within the sequences are not structurally superposable. Such positions, often in the loop regions, can be significant in determining function.

Protein superfamilies provide distinct challenges to phylogenetic reconstruction. Following gene duplication, proteins can undergo significant structural and functional changes associated with neofunctionalization, resulting in loop regions and other parts of protein structures not being strictly homologous across all members of a multigene family (see Figure 2). Even among orthologs, evolutionary rates can vary greatly within different lineages [27,28]. This degree of extreme structural and sequence diversity clearly violates the assumptions of most simple (and therefore computationally tractable) models of evolution.

Assessing the expected accuracy of phylogenetic methods for protein superfamily reconstruction is a challenge in itself. Unlike phylogenetic reconstruction of species trees, where fossil evidence exists to help investigators assess tree accuracy, we have no fossil record for protein superfamilies. Simulation studies have tended to assume models of molecular evolution that are appropriate to single orthologous DNA sequences [29], but do not normally address many of the complexities of protein multigene family evolution. This has begun to change; models have been introduced that incorporate a wider range of information, such as indel evolution and structural constraints [30–33]. Still, we believe there is a long way to go in this regard before simulation studies can effectively assess the expected accuracy of phylogenetic inference in protein superfamilies.

An additional complication in phylogenetic reconstruction of protein families is the almost universal dependence on an accurate MSA as input. Studies of alignment accuracy for pairs of proteins at different levels of evolutionary and structural divergence show dramatic increases in alignment errors with sequence divergence [34]. Several recent methods have bypassed this issue by concurrent estimation of a phylogeny and an MSA from unaligned sequences [35–37]; we look forward to future developments in this area.

Another barrier to the use of phylogenomic inference methods is their computational complexity. Owing to the large size of protein superfamilies (with hundreds or thousands of taxa), many applications of phylogenomic inference employ fast distance-based methods instead of character-based approaches or forego even simple models of evolution in favor of faster hierarchical clustering algorithms (e.g., the Panther system [38]). Without an objective understanding of the expected accuracy of individual phylogenetic tree estimation methods under different conditions, we cannot know whether functional inferences based on these analyses are accurate.

In practice, assessing the likely accuracy of a particular tree is typically accomplished through bootstrap analysis or comparison of trees constructed using different phylogenetic reconstruction methods. Analysis of multiple trees for a given family often shows regions of agreement as well as significant differences of opinion: closely related subtrees are often found consistently across different methods, with primary differences between trees being at the coarse branching order between these conserved subtrees. Functional inferences can then be based on subtrees with high bootstrap support or on those subtrees that are found in the strict or majority consensus of several tree methods. However, these methods of analysis are quite time consuming and impractical for large datasets or for high-throughput application.

## The Reliability and Source of Existing Database Annotations

Any system of functional inference depends on the accuracy of the characterized members. The Gene Ontology Consortium has provided a mechanism whereby sequence annotations have associated *evidence codes,* documenting the origin of the annotation (e.g., by electronic means, by direct assay, or by a traceable author statement) [39]. We believe that annotation transfer, even in a phylogenomic context, should only be performed when solid *experimental* support is available. Our analysis of more than 300,000 proteins in the UniProt database shows only 3% of proteins with functional annotations have experimental support. We suspect that many more proteins than these have been experimentally pursued, but that the results of these experiments are not being propagated efficiently (or at all) to the sequence databases or to the GO Annotation project [40]. One reason for this is the lack of proper usage of standard sequence identifiers in the biological literature, and we applaud the efforts at various journals to improve this status quo (see, e.g., *Genome Research* and the PLoS journals). We would go further and recommend that sequence databases specifically encourage ontology annotation during sequence submission. We expect that advances in text-mining software will also help correct the imbalance, although the field is not yet at a point to contribute on a large scale [41]. Finally, we believe that mechanisms must be put into place to enable annotation errors to be more easily corrected. The UniProt database responds to community requests for annotation error correction; other sequence databases might do well to follow their lead.

## Functional Inference Based on Assumed Orthology

Orthologs—genes or proteins related by speciation—are generally assumed to have greater functional similarity than paralogs, which are related by gene duplication. However, inference accuracy also depends on evolutionary distance and the particular functional attribute under consideration. Some attributes of protein families, such as the three-dimensional structure, persist across large evolutionary distances. Other attributes, such as substrate specificity, can be modified based on a handful of amino acid substitutions in critical positions. The persistence of certain traits may be more limited in some families and more expansive in others. The assumption that orthology implies a functional similarity must therefore be tempered by an assessment of evolutionary distance [42,43].

Moreover, determining orthology is not always straightforward. RIO and Orthostrapper take the approach of using phylogenetic trees to assess orthology between homologs [14,15]. This is clearly the most accurate method, although accuracy will depend on the estimated phylogeny. However, these methods require estimation of a new tree for each family of interest, and trees must be recomputed whenever novel sequences are added to the family. This limits their application in large-scale endeavors. The COG database makes the simplifying assumption that proteins are orthologs if they are reciprocal top BLAST hits [13], but this limits the resulting relationships, and domain-shuffling, high sequence diversity within the family, and incomplete genome sequencing can all contribute to error.

Finally, the dearth of experimental evidence supporting functional annotations, together with ambiguous tree topology reconstruction, often limits the number of proteins that can be annotated effectively based strictly on orthology. Because of the limitations in restricting functional annotations to orthologs, methods have been developed to allow functional inference to extend beyond the strict confines of orthology. The SIFTER algorithm enables annotations to be propagated over a phylogenetic tree, using GO annotations and priors over existing annotations [22]. We believe this Bayesian approach shows great promise in automating the functional annotation of novel sequences.

## The Future of Phylogenomic Inference

We have focused in this paper on the use of phylogenomic inference of protein function. However, phylogenomic inference can be applied to a wide array of protein family attributes. Selection of templates for comparative model construction can be performed in a phylogenomic context, e.g., picking the template that has the smallest tree distance to a target of unknown structure. Phylogenomic inference of pathway involvement may also be possible under some circumstances, for instance, in cases in which a subtree contains orthologs in closely related species.

Looking to the future of phylogenomic analysis, we believe that the greatest improvement to this field will take place when investigators have access to rigorously validated biological data through which phylogenomic methods can be assessed for accuracy. The Structure Function Linkage Database [44], which links protein structures with detailed information on partial chemical reactions, is an important contribution in this regard. Carefully designed benchmark datasets, such as those developed by the protein structure prediction community (e.g., the Astral datasets [45] and SCOP [46]), as well as the international biennial CASP experiment [47], have the potential to transform the field. The protein structure prediction field is one of the most mature in all of computational biology, and we believe this is due (at least in part) to the availability of challenging benchmark datasets and international experiments. The phylogenomic community needs analogous datasets appropriate for our own development and maturation. The natural competitiveness of computational biologists is used to good measure when we can push our methods to ever-increasing levels of accuracy. 

## Abbreviations

HMM: hidden Markov model
MSA: multiple sequence alignment
SCOP: Structural Classification of Proteins
